# Niobium-Based Conditioning Layer to Reduce Bacterial Adhesion and Biofilm Formation on Titanium Surface

**DOI:** 10.1021/acsomega.5c12932

**Published:** 2026-03-26

**Authors:** Viviane C. Oliveira, Nilza L. Magalhães, Carla R. O. Maciel, André F. A. S. Silva, Lucas L. Bim, Carolina Chaves, Cássio do Nascimento, Francisco W. Paula-Silva, Cláudia H. Silva-Lovato, Ana P. Ramos, Adriano M. Ferreira, Evandro Watanabe

**Affiliations:** † Departamento de Materiais Dentários e Prótese, Faculdade de Odontologia de Ribeirão Preto, 67784 Universidade de São Paulo, Ribeirão Preto 14040-904, São Paulo, Brazil; ‡ Centro de Investigación Biomédica en Red de Enfermedades Respiratorias (Ciberes), Hospital Clinic de Barcelona, Barcelona 08036, Spain; § Departamento de Clínica Infantil, Faculdade de Odontologia de Ribeirão Preto, Universidade de São Paulo, Ribeirão Preto 258-9654, São Paulo, Brazil; ∥ Departamento de Enfermagem Geral e Especializada, Escola de Enfermagem de Ribeirão Preto, Universidade de São Paulo, Ribeirão Preto 258-9654, São Paulo, Brazil; ⊥ Departamento de Química, Faculdade de Filosofia, Ciências e Letras de Ribeirão Preto, Universidade de São Paulo, Ribeirão Preto 258-9654, São Paulo, Brazil; # Fundação Santa Lydia, Ribeirão Preto 14085-070, São Paulo, Brazil; ∇ Egas Moniz School of Health & Science, Instituto Universitário Egas Moniz, Almada 2829-511, Portugal; ○ 54534 Universidade Federal de Mato Grosso do Sul, Três Lagoas 79070-900, Mato Grosso do Sul, Brazil; ◆ Departamento de Biologia Básica e Oral, Faculdade de Odontologia de Ribeirão Preto, Universidade de São Paulo, Ribeirão Preto 258-9654, São Paulo, Brazil

## Abstract

Niobium metal has a wide range of applications; however, the development of Nb-coated surfaces with antimicrobial activity remains unexplored. This study investigates the antimicrobial and antibiofilm activities of ammoniacal niobium oxalate (ANO) and develops a methodology to deposit it on titanium-functionalized surfaces to prevent bacterial colonization and biofilm formation. ANO is dispersed in water and characterized for particle size, Fourier transform infrared spectroscopy, X-ray diffraction, ζ-potential, and *in vitro* cytotoxicity. Its antimicrobial activity is assessed by microdilution and inhibition halo assays against *Enterococcus faecalis*, *Escherichia coli*, *Pseudomonas aeruginosa*, methicillin-resistant *Staphylococcus aureus*, *Staphylococcus epidermidis*, *Streptococcus mutans*, *Candida albicans*, and *Candida glabrata*. Antibiofilm activity is evaluated through biomass quantification, respiratory activity, and morphological analysis. Titanium surfaces functionalized with polyacrylic-acid–ANO films are tested under dynamic flow conditions for their antifouling properties in a multispecies biofilm model. ANO particles (∼450 nm) exhibit a negative charge, high crystallinity, and low cytotoxicity. The compound inhibits both Gram-negative and Gram-positive bacteria, even at low concentrations, and reduces the metabolic activity of mature biofilms. However, it does not remove aggregates or prevent adhesion and biofilm growth on titanium surfaces, indicating the need for further optimization of the functionalization conditions.

## Graphical abstract

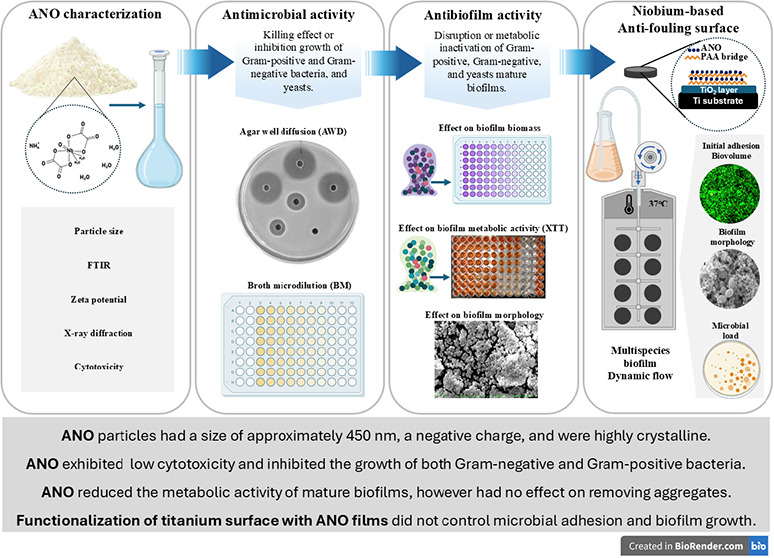

## 1 Introduction

Since the discovery of niobium (Nb), scientific advancements in its application have been vast and transformative, with potential applications spanning a wide range of fields. Currently, the metal is present in steels, superalloys, intermediate materials, and metal alloys, as well as in compounds, coatings, nanomaterials, optoelectronic devices, and catalysts.^1,2^ As a result of the complete niobium production chain, from mineral extraction and beneficiation to refining and alloy production, different subproducts are generated, including niobium oxides, ferroniobium, niobium phosphate, niobic acid, ammonium niobium oxalate, and niobium carbide, among others.^1^ Extensive research has demonstrated diverse applications of pure Nb and its subproducts, including their relevance in metallurgy, electronics, and most recently in the biologic field.^2–5^

Regarding the biological properties of niobium-based compounds, there are numerous reports suggesting good bioactivity, elevated biocompatibility, and corrosion resistance.^2,3,6–10^ For instance, the use of a bioactive glass containing niobium oxide (Nb_2_O_5_) or niobium chloride (NbCl_5_) promoted positive effects on bone healing by inducing the proliferation of osteoblasts, favoring the osteogenesis.^6,7^ In the area of wound healing, a hydrogel dressing with niobium carbide (Nb_2_C) showed good biocompatibility and reduced toxicity.^8^ In association with photothermal therapy, another dressing containing niobium pentoxide (Nb_2_O_5_) demonstrated a positive effect on wound healing.^9^ In the dentistry area, an experimental orthodontic adhesive containing Nb_2_O_5_ was able to induce mineral deposition,^10^ and a niobium phosphate (Nb_4_PO) bioglass also demonstrated inhibition of the dental demineralization process.^11^

Besides the biological activity, the antimicrobial activity of niobium-based compounds against different Gram-positive and Gram-negative bacteria is evident.^8–11^ Of utmost importance, Nb ions (Nb+HNO_3_) had bactericidal action against *Acinetobacter baumannii* and *Klebsiella pneumoniae*, microorganisms belonging to the ESKAPE group.^12^ The combination with other metals exhibited a synergistic effect, demonstrating a reduction in biofilm formation after incorporating Nb with copper (Cu^+^) and silver (Ag^+^).^13,14^ Additionally, there is evidence of low toxicity of niobium-based compounds, indicating broad possibilities for applying them as antimicrobial agents, including surface coatings.^3,4,15^

Coatings and surface modifications based on other metal ions, such as Ag^+^, Cu^+^, or zinc (Zn^2+^), have well-known antimicrobial properties and are widely used in various applications, such as medical devices and packaging.^16–18^ Generally, these agents affect the integrity of the cell membrane, generate reactive oxygen species (ROS), inhibit enzymatic activity, or damage biomolecules, interfering with the viability and replication of microorganisms. However, despite the wide range of Nb-based compounds, there is a noticeable scarcity of research exploring their antimicrobial and antibiofilm activity as coating layers, leaving gaps in their potential biomedical applications as bioactive materials.

Given the biological characteristics of Nb-based compounds and their promising antimicrobial activity, this study characterized and evaluated the cytotoxicity, antimicrobial, and antibiofilm activities of Ammoniacal Niobium Oxalate (ANO) (NH_4_[NbO]­(C_2_O_4_)_2_). Concurrently, the antibiofilm activity of titanium (Ti) surface, functionalized with ANO films, was evaluated.

## 2 Results and Discussion

### 2.1 ANO Characterization

In this study, the antimicrobial and antibiofilm properties of the ANO compound were investigated. In parallel, Ti surfaces functionalized with ANO were developed, with the aim to reduce microbial adhesion and biofilm formation, with potential applications in medical and dental fields. The findings indicate that ANO has marked antimicrobial activity and reduces the metabolic activity of biofilms; however, it does not remove microbial aggregates. Additionally, Ti surfaces functionalized with ANO showed no reduction in microbial adhesion or biofilm formation when tested under continuous-flow conditions, which mimic implant-associated environments.

ANO was selected to evaluate the antimicrobial and antibiofilm activities of the Nb metal ion because of its high water solubility. The presence of oxalate and ammonium ions contributes to this solubility, enabling the compound to disperse readily in aqueous solutions and facilitate its application in chemical processes such as thin-film deposition or niobium-based syntheses.^11,19^ In fact, upon dispersion, the mean hydrodynamic diameter of ANO particles was 450 nm, as determined by the peak of the size distribution (Figure 1A).

**Figure 1:**
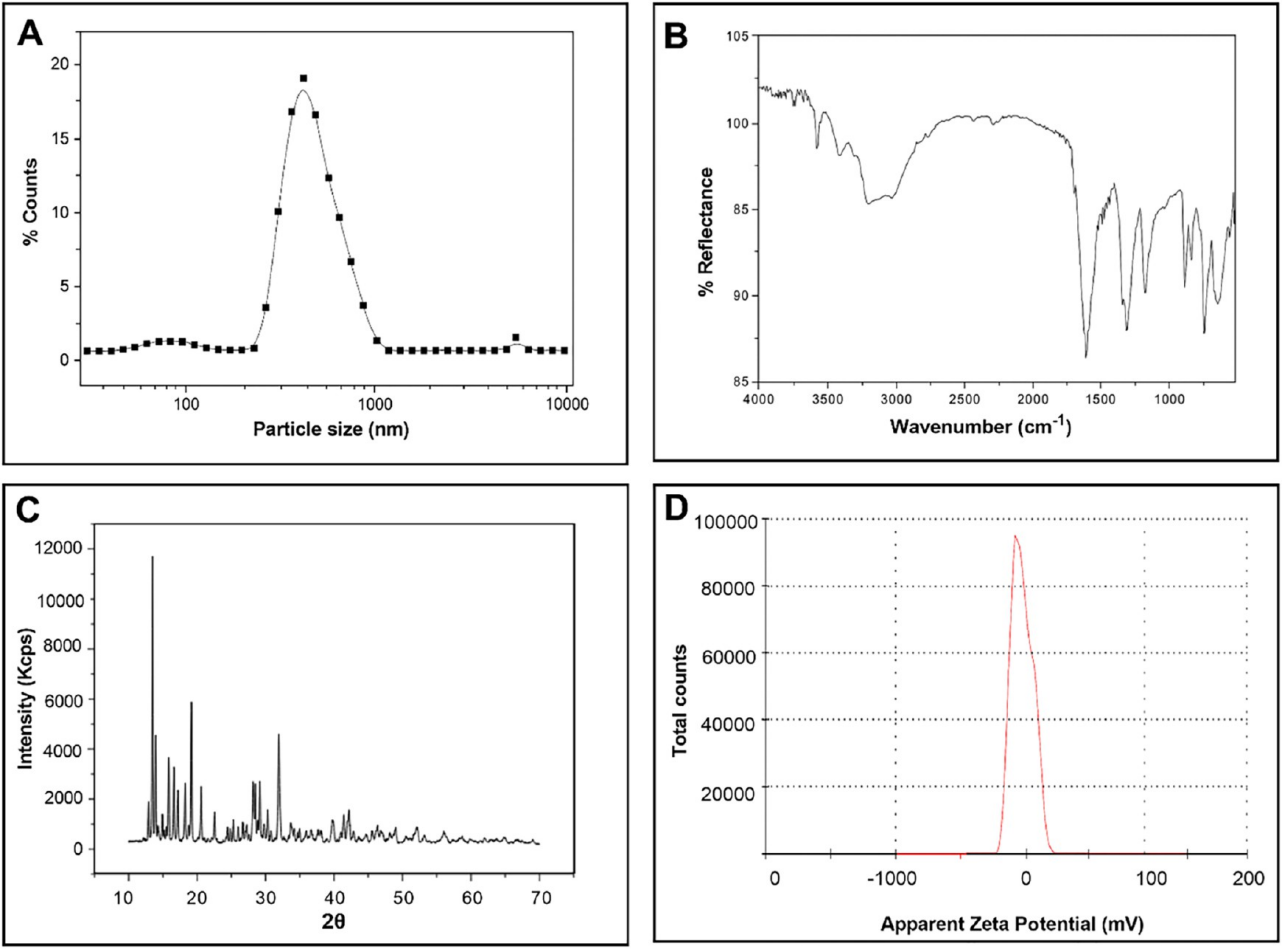
Characterization of the compound ANO after dispersion in water. (A) Size distribution of ANO particles, (B) Fourier transform infrared spectroscopy (FTIR), (C) X-ray diffraction, (D) ζ-potential distribution.

The presence of niobium oxide can be identified by characteristic νNb=O in the 500–1000 cm^–1^ range. In addition, the spectrum exhibits intense bands between 1600 and 1400 cm^–1^, which arise from the stretching vibrations of the carboxylate groups (C=O and C–O bonds) of the coordinated oxalate ligands. A broad absorption band around 3000–3400 cm^–1^ indicates the presence of hydroxyl groups and ammonium ions through νO–H and νN–H vibrations. These features confirm the molecular structure of ammoniacal niobium oxalate, comprising Nb=O units coordinated to oxalate ligands and stabilized by NH_4_^+^ counterions (Figure 1B).^19^ The X-ray diffraction pattern of the particles revealed that they are composed of high crystalline ammonium niobium oxalate (Figure 1C).^20^

The ζ-potential (mV) close to zero indicates low colloidal stability of the dispersions, which may cause aggregation or agglomeration (Figure 1D), in part justifying the broad size distribution observed in Figure 1A.

### 2.2 Antimicrobial Activity of ANO

Antimicrobial activity was defined as either a killing effect or growth inhibition against Gram-positive and Gram-negative bacteria and yeasts. ANO efficiently inhibited the growth of clinically relevant Gram-positive and Gram-negative bacteria at low concentration (MIC ranged from 1.56 to 6.25 mg mL^–1^; MBC ranged from 6.25 to 25 mg mL^–1^). However, the compound did not show the same activity against yeasts. For *C. albicans* and *C. glabrata*, the MIC values were higher, and the MFC were out of the evaluated range (Figure 2).

**Figure 2:**
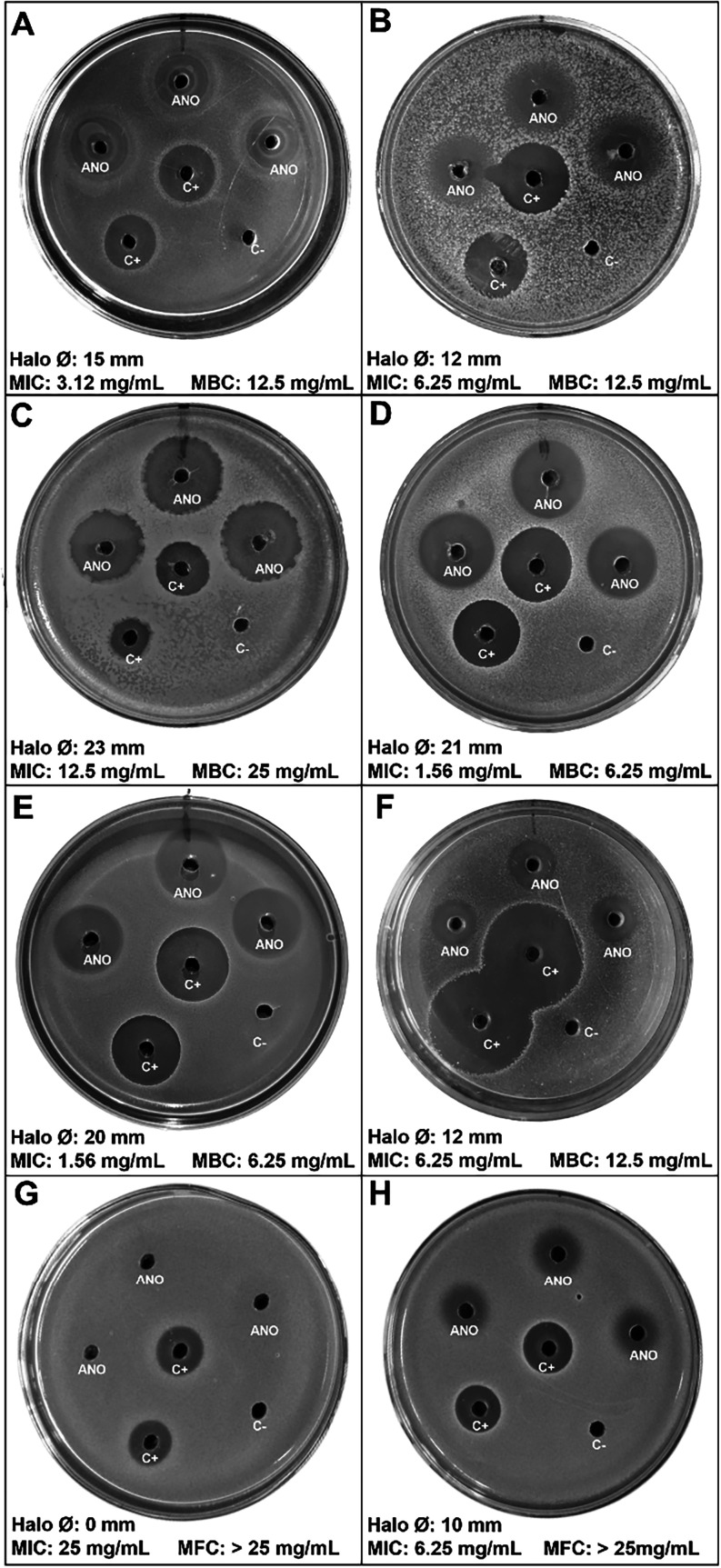
Well diffusion assay demonstrating the antimicrobial activity of ANO. (A) *E. faecalis*; (B) *E. coli*; (C) *P. aeruginosa*; (D) MRSa; (E) *S. epidermidis*; (F) *S. mutans*; (G) *C. albicans*; (H) *C. glabrata*. ANO: 200 mg mL^–1^; Positive control: Chlorhexidine 0.12%; Negative control: PBS. The minimum inhibitory concentration (MIC), minimum bactericidal concentration (MBC), and minimum fungicidal concentration (MFC) were determined through the broth microdilution assay. The results are descriptive, and no statistical comparisons were carried out.

ANO inhibited the planktonic growth of Gram-negative and Gram-positive bacteria and yeasts. The microorganisms included in this study are causes of severe healthcare-associated infections (HAIs), such as pneumonia, urinary tract infections, and bloodstream infections, which highlights the importance of our findings.^21–23^ Although the exact mechanism that confers Nb its antibacterial effect is not known, it is suggested that metal ions interfere with essential cellular processes, such as (i) Generation of ROS, causing oxidative stress; (ii) Interaction with the cell membrane, impacting microbial cell integrity; (iii) Interaction with nucleic acids, primarily destabilizing the DNA molecule; and (iv) Inhibition of enzymatic activity, especially enzymes involved in energy production and cell growth.^24,25^

### 2.3 Antibiofilm Activity of ANO

Antibiofilm activity was defined as biofilm disruption or metabolic inactivation in biofilms formed by Gram-positive and Gram-negative bacteria and yeasts.

The immersion of mature biofilms in different concentrations of ANO drastically reduced the metabolic activity of the bacteria. The reduction was equivalent to that of the positive control (sodium hypochlorite). However, in biofilms formed by *C. albicans* and *C. glabrata*, the reduction was less evident and dependent on a higher concentration of ANO (Figure 3). For *P. aeruginosa* and *S. mutans*, the reduction was time-dependent, as increasing the contact time resulted in an evident inhibition of metabolic activity. Despite the reduction in metabolic activity, the compound had no effect on biofilm biomass. In comparison to the negative control, residual biofilm aggregates remained unchanged, indicating an inability to remove dead biofilm (Figure 3).

**Figure 3:**
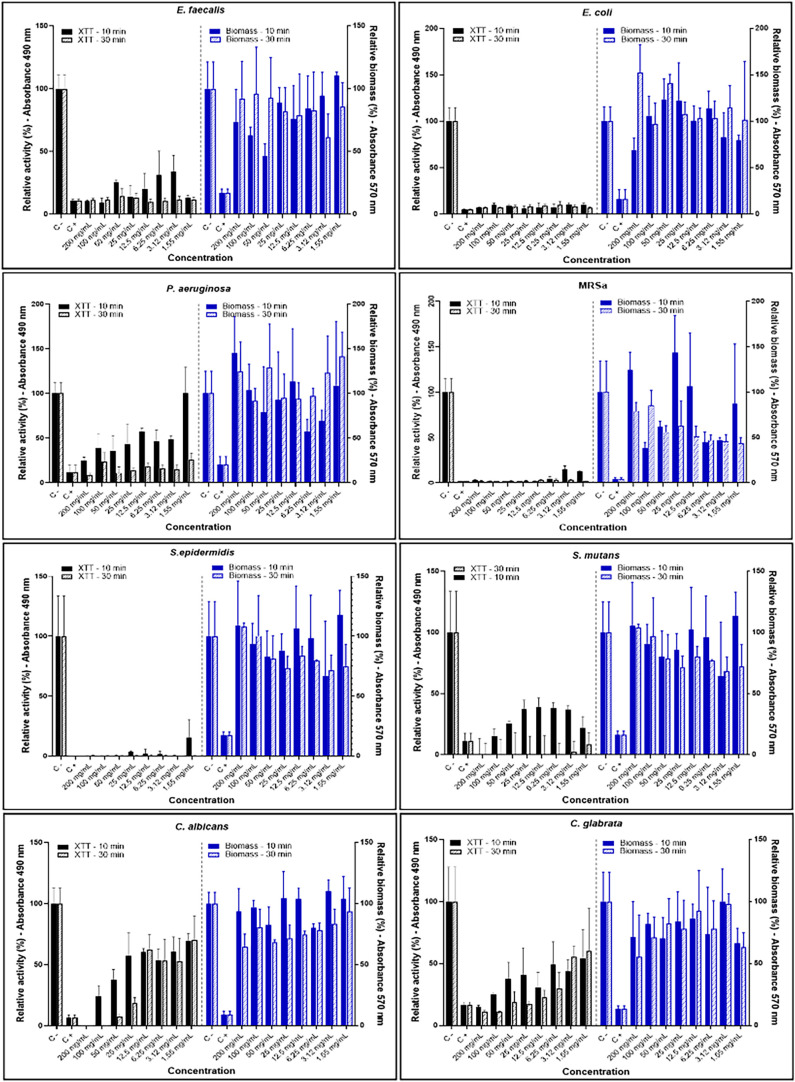
Metabolic activity [absorbance at 492 nm (right *y* axis)] and biofilm biomass [absorbance at 570 nm (left *y* axis)] (mean ± standard deviation) of *E. faecalis*, *E. coli*, *P. aeruginosa*, MRSa, *S. epidermidis*, *S. mutans*, *C. albicans*, and *C. glabrata* mature biofilms, after immersion in different concentrations of ANO during 10 and 30 min. The results are presented as the activity relative to the negative control (immersion in PBS). The results are descriptive, and no statistical comparisons were carried out.

The morphological evaluation of biofilms revealed a huge colonization of surfaces, without noticeable differences between the samples treated and untreated with ANO. The analysis revealed the presence of dense and organized layers of Gram-positive bacteria, Gram-negative bacteria, and yeasts, illustrating a 3D structure to the biofilm (Figure 4).

**Figure 4:**
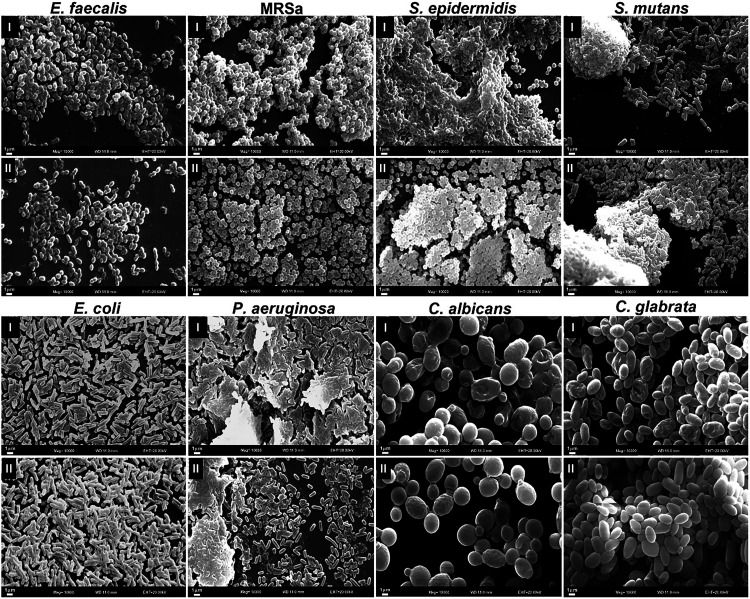
Scanning electron micrographs of *E. faecalis*, *E. coli*, *P. aeruginosa*, MRSa, *S. epidermidis*, *S. mutans*, *C. albicans*, and *C. glabrata*. (I) Biofilms after immersion in ANO for 30 min. (II) Biofilms after immersion in PBS (Control). Magnification 10,000×. Scale bar = 1 μm.

To explore the mechanism of action, biofilms were examined by SEM after immersion in ANO. No morphological alterations indicative of membrane damage were observed in any of the microorganisms evaluated. However, a significant reduction in the metabolic activity of mature biofilms was detected. Together, these findings suggest that ANO does not primarily disrupt the cell structure but may interfere with cellular function. We therefore hypothesize that ANO interacts with microbial cells and inhibits enzymatic activity, as previously described for other metal ions.^24,25^ Additionally, after dispersion in water, ANO exhibited an acidic pH, which may contribute to protein and enzyme denaturation, thereby impairing microbial metabolism. Consistent with the inhibition of planktonic growth, the absence of structural damage and the lack of biofilm disruption were accompanied by no reduction in the total biofilm biomass. Collectively, these observations support a model in which ANO predominantly affects the metabolic activity rather than biofilm integrity. It is important to note that an effective antibiofilm agent should eliminate microbial aggregates, since residual biofouling promotes increased biomass and thickness, facilitating subsequent surface recolonization.^26^

### 2.4 Characterization of the ANO-Based Conditioning Layer

The slight variation in the intensity of the bands in the ATR-FTIR spectra of the samples containing 8, 12, and 16 layers indicates that after 8 deposition cycles, the films reached a constant amount of deposited material (Figure 5A). Therefore, 8 deposition cycles were used in the subsequent steps to prepare the ANO-based conditioning layer. The FTIR spectrum of the films deposited with PAA–ANO exhibited characteristic bands of carboxylic groups in 1700–1600 cm^–1^ (νC=O) and 1450–1380 cm^–1^ (νCOO^–^), along with intense absorptions in the 1300–1100 cm^–1^ region, attributed to vibrational modes of oxalate coordinated to niobium. Moreover, the absence of the broad band close to 3400 cm^–1^ and the presence of a narrow band at 3800 cm^–1^ are related to the presence of structured hydroxyl groups, revealing organization of the coating. The 800–600 cm^–1^ region showed vibrations associated with Nb–O and Nb=O bonds, suggesting an environment that may arise from both coordinated niobium–oxalate species and partial formation of niobium–oxide structures.^20^ The titanium-functionalized surface was then characterized by using SEM. A nonhomogeneous film was observed on the treated surface, with drop-like coating. Overall, ATR-FTIR spectra and SEM evaluation confirmed the efficiency of the proposed protocol (Figure 5B,C).

**Figure 5:**
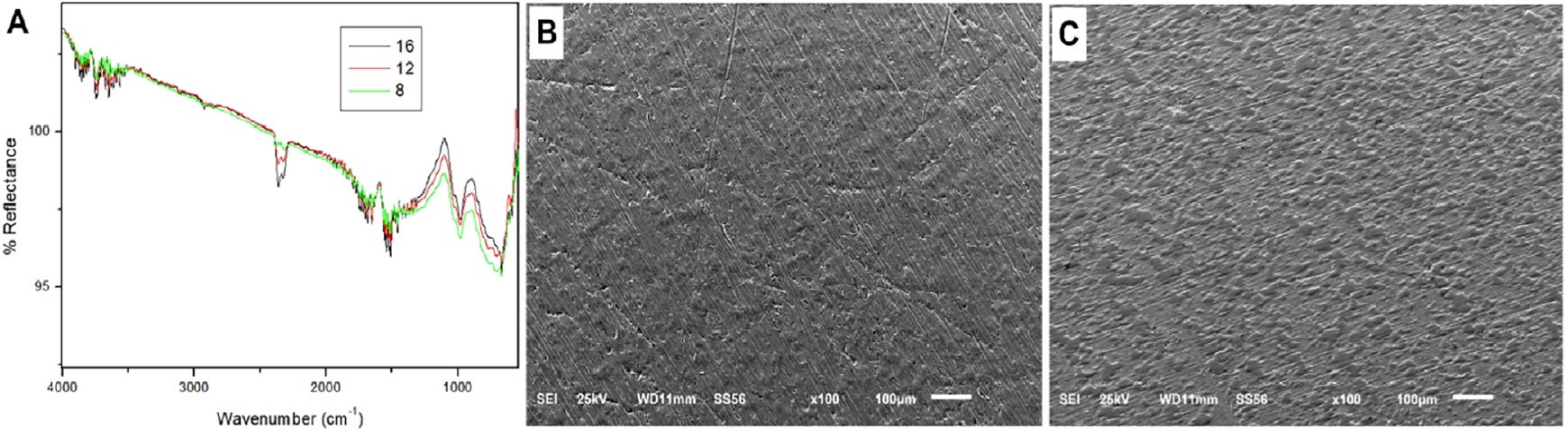
Characterization of the ANO-based conditioning layer. (A) Fourier transform infrared spectroscopy (FTIR) after 8, 12, and 16 sequential deposition cycles. (B) Scanning electron micrographs of a Ti specimen without covering (Control) and (C) Ti specimen covered with the PAA-ANO film. Magnification 1000×. Scale bar = 100 μm.

### 2.5 Cytotoxicity of ANO and ANO-Based Conditioning Layer

Eukaryotic cells treated for 24 h with ANO at concentrations ranging from 1.25 to 5.0 mg mL^–1^ maintained their ability to metabolize MTT salt, indicating low compound toxicity (Figure 6A). Although the difference was not statistically significant, it is possible to observe concentration-dependent toxicity. Similarly, the surface functionalized with ANO at 5.0 mg mL^–1^ did not promote significant alterations in cell viability compared to the control (Figure 6B), suggesting the biocompatibility of the ANO-based conditioning layer.

**Figure 6:**
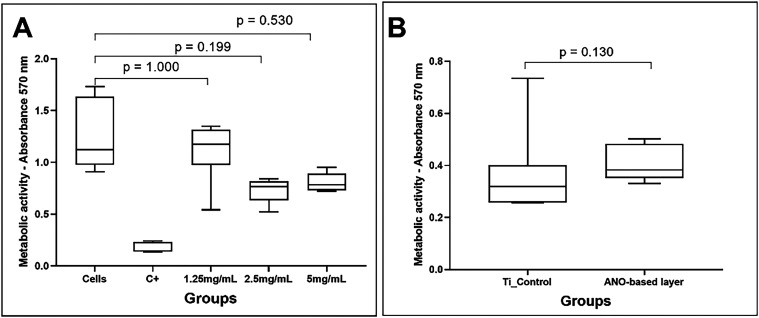
Cytotoxicity of ANO and ANO-based conditioning layer in L929 cells. (A) Assay carried out in cells adhered to the bottom of a 96-well plate. Comparisons among groups were conducted through the Kruskal–Wallis test followed by the Dunn post hoc test with Bonferroni adjustment. (B) Assay carried out in cells cultured on the surface of control and experimental specimens. Comparisons among groups were conducted through the Mann–Whitney test.

ANO exhibited low cytotoxicity, even in its pure form, corroborating previous findings in the scientific literature. Dsouki et al. treated mice for up to 12 days with niobium pentoxide (Nb_2_O_5_) at 3%.^4^ The authors showed no progressive hepatotoxicity and no hematological alterations, indicating that the metal Nb is a promising alternative for biomedical applications. Indeed, the scarcity of data evaluating ANO compromises direct comparison. Most studies involving the metallic ion employ Nb_2_O_5_, which is the most extensively investigated oxide.^27^ Recently, Kolya and Kang emphasized the need for comprehensive studies to fully understand the potential implications for human health regarding exposure to metal oxides.^28^ We consider it essential to further investigate the biocompatibility of ANO-based coatings. Beyond antibiofilm performance, several additional factors are critical for assessing their translational potential in the biomedical field, including cytocompatibility with different cell types under varied incubation conditions, associated cell death mechanisms, corrosion behavior, mechanical stability, and long-term surface performance.^27^

### 2.6 Antifouling Activity of the Functionalized Surface

Antifouling activity was defined as the ability of a surface to inhibit microbial adhesion and early biofilm formation.

The area (μm^2^) covered by live (Figure 7A) and dead (Figure 7B) microorganisms was similar between those of the functionalized and control surfaces. As expected, a progressive increase in the area covered by live microorganisms was observed over time; however, adhesion performance did not significantly differ across surfaces after 1 h (Figure 7C), 2 h (Figure 7D), and 4 h (Figure 7E) under dynamic flow conditions.

**Figure 7:**
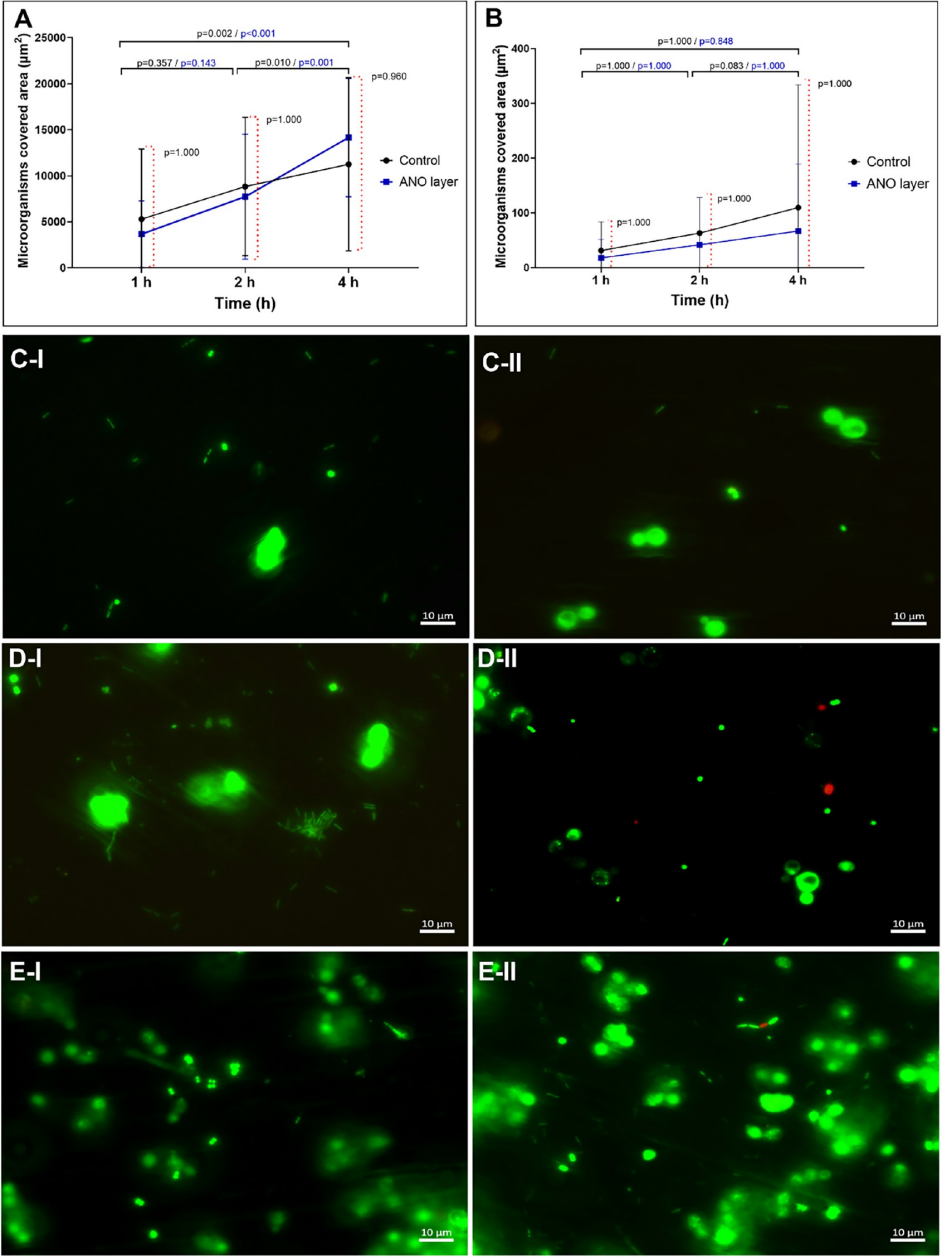
Microorganisms’ adhesion to functionalized surface. (A) Area (μm^2^) covered by live microorganisms. (B) Area (μm^2^) covered by dead microorganisms. Horizontal brackets indicate within-group comparisons across time. Red vertical dashed brackets indicate between-group comparisons at each time point. Data were analyzed using a generalized linear model with multiple comparisons and Bonferroni adjustment. (C–E) Fluorescent micrographs of the evolution of microorganisms’ adhesion on ANO-based (I) and control (II) surfaces after 1 h (C), 2 h (D), and 4 h (E) of flow.

Notably, biofouling process is influenced by material properties, and modifications to surface characteristics such as roughness and surface free energy can significantly affect biofilm formation.^29^ These characteristics can increase or decrease the propensity of bacteria to adhere to the surface, which has important implications for the control of infections associated with medical devices. Thus, this study proposed the development of an experimental surface coated with an ANO film, aiming to advance antifouling Ti surfaces for application in medical and dental devices, such as implants and surgical instruments.

Ti, when exposed to air, forms a layer of titanium oxide (TiO_2_),^30^ which can chemically interact with PAA, creating a hydrophilic surface that facilitates the formation of thin films.^31^ In this study, PAA was used as a bridge between Ti and ANO (TiO_2_–PAA-ANO), ensuring the adhesion of Nb to the Ti surface. The objective was to form an experimental surface of PAA-ANO as a protective and functional layer with antifouling activity. The electrodeposition of Nb_2_O_5_ on a nickel–titanium (NiTi) alloy from aqueous electrolytes has previously been demonstrated by Safavi et al.^32^ The authors reported that the film on the alloy surface significantly reduced the adhesion of *E. coli* and *S. aureus*.

No difference was observed in the microbial load of *S. aureus* (*p* = 0.716), *C. albicans* (*p* = 0.094), and *P. aeruginosa* (*p* = 0.086) after 24 h of biofilm growth (Figure 8A). Regarding biovolume (μm^3^), both surfaces showed similar amounts of live (*p* = 1.000) and total biofilm (*p* = 0.809). However, the amount of dead biofilm was significantly higher on the ANO-functionalized surface compared to control (*p* < 0.001) (Figure 8B).

**Figure 8:**
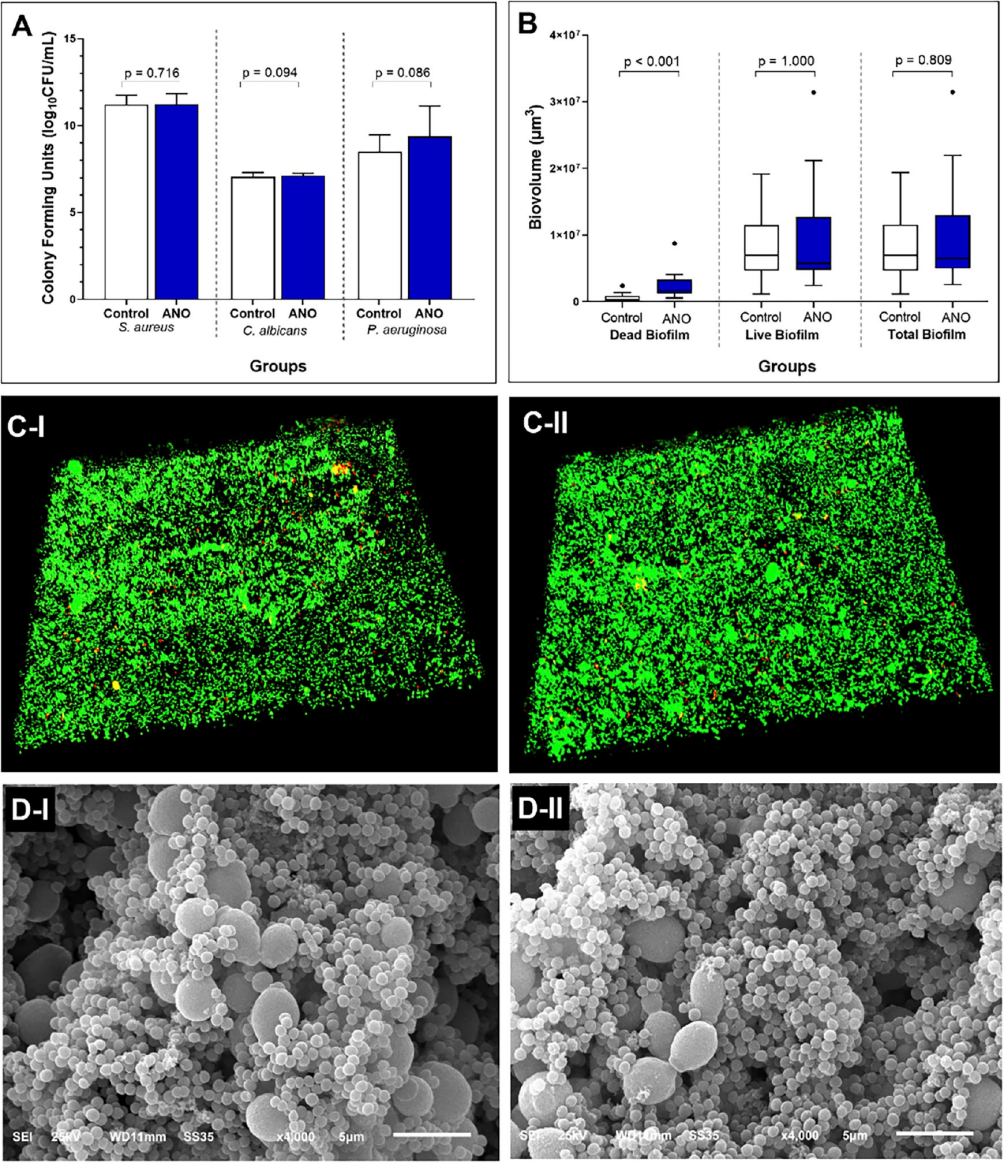
Antibiofilm activity of the functionalized surface. (A) Microbial load (log_10_ CFU mL^–1^) of *S. aureus*, *C. albicans*, and *P. aeruginosa* on control and experimental surfaces. Data sets from control and experimental surfaces were compared using the Student’s *t* test. (B) Quantification (μm^3^) of dead, live, and total biofilm on control and experimental surfaces. Data were analyzed using the Mann–Whitney test. (C, D) Representative 3D and SEM micrographs of multispecies biofilm grown on ANO-based (I) and control (II) surfaces after 24 h of flow.

CLSM and SEM images highlight a dense biofilm layer adhered to both surfaces, structurally organized in multiple layers (Figure 8C,D). Qualitatively, the morphological characteristics of the three evaluated microorganisms could be identified, with no significant differences between the functionalized and control surfaces.

Here, although the presence of an ANO-based conditioning layer was confirmed - evidenced by SEM micrograph and FTIR detection of Nb–O bonds (Figure 5), no significant reduction in the adhesion or biofilm formation of *C. albicans*, *S. aureus*, and *P. aeruginosa* was observed. Only an increase in the number of dead cells was noted after 24 h of incubation on the surface functionalized with ANO. The higher number of dead cells may be associated with the ability of ANO to alter cell metabolism and induce cell death in direct contact with the functionalized surface, without necessarily impacting the adhesion of microorganisms in adjacent layers. We hypothesize that the action of the compound could be localized, limited to the cells closest to the TiO_2_–PAA-ANO interface, while microorganisms in the upper layers of the biofilm remain protected. Contradictorily, Souza et al., when evaluating the incorporation of Nb_2_O_5_ in an experimental composite, showed lower biofilm formation, lower total biofilm biomass, and a higher percentage of dead cells.^33^ However, it is important to consider that ANO has not been extensively evaluated, and given the biofilm formation model employed, leaching of the PAA–ANO film may have occurred, which could have compromised the effectiveness of the experimental surface in inhibiting adhesion and biofilm formation. This leaching may have resulted in the gradual release of the PAA-ANO film, reducing its antimicrobial potential over time, which could explain the lack of a clear reduction in biofilm formation observed in this study.

Niobium-containing layers can be produced using several techniques, including sputter deposition, sol–gel processing, annealing of CaP-coated niobium-containing substrates, and air plasma spraying.^27,32^ Our findings suggest that the use of polymeric compounds to produce niobium oxide–containing coatings may offer certain advantages; however, further optimization would be required before considering biomedical applications. Importantly, Detomaso et al. demonstrated that polymeric coatings based on PAA, when noncovalently attached, are susceptible to release and leaching under aqueous or flow conditions, leading to a loss of functionality and might not be considered for long-term cell culture.^34^

Finally, some potential limitations should be considered in this study. The leaching of the ANO film was not evaluated, which could provide crucial information about the durability and effectiveness of the compound over time. Additionally, the lack of data on the amount of Nb in the compound makes it difficult to establish a direct interpretation regarding the antimicrobial activity and biofilm control capacity of the metallic ion. Future studies should focus on biocompatibility of ANO coating, as well as different deposition methods to optimize the effectiveness of the compound as an antifouling agent.

## 3 Conclusion

Given the experimental conditions, it is concluded that ANO presented significant antimicrobial activity against planktonic microorganisms and was able to reduce the metabolic activity of mature biofilms. However, the functionalization of Ti surfaces with PAA-ANO had no effect on reducing microbial adhesion or inhibiting biofilm growth.

## 4 Experimental Section

### 4.1 ANO Characterization

The compound ANO presented itself as a water-soluble white powder. According to the manufacturer (Companhia Brasileira de Metalurgia e Mineração-CBMM, Araxá, MG, Brazil), the niobium content (Nb_2_O_5_) is about 23 wt % by weight, and its solubility in water is 430 mg mL^–1^ at 20 °C.

The stock solution was prepared by dispersing 10 g of ANO in 50 mL of ultrapure water (resistivity 18.2 MΩ cm and surface tension 72.13 mN m^–1^ at 25 °C), followed by homogenization in an ultrasonic bath (Altsonic, Ribeirão Preto, SP, Brazil) for 10 min. The dispersed compounds were then characterized.

Fourier transform infrared spectroscopy (FTIR) was employed to identify the chemical groups present in the samples. Measurements were performed using an IRPrestige-21 spectrophotometer (Shimadzu, Tokyo, Japan) equipped with an Attenuated Total Reflectance (ATR) accessory. Samples were placed directly onto the ATR crystal, ensuring a uniform contact. Spectra were collected in the range of 4000–400 cm^–1^. The absorption bands were assigned to functional groups according to the characteristic vibrational frequencies.

The crystallinity of the compound was studied by X-ray diffraction (XRD) on a diffractometer (AXS D5005; Bruker, Karlsruhe, Germany) operating with Cu Kα radiation at a grazing incidence angle. The diffraction data were collected over a 2θ range of 10–70°.

The hydrodynamic size distribution and ζ-potential of ANO were measured using a Zetasizer Nano ZS (scattering angle of 170°, 580 nm laser, Malvern Instruments, Malvern, U.K.) by dynamic light scattering (DLS) and electrophoretic light scattering (ELS). Measurements were performed at room temperature, and the reported values represent the average of three independent measurements.

The working solutions were prepared before using by diluting the stock solution in ultrapure water or culture medium. The stock solution was used for no longer than 3 days after dispersion.

### 4.2 Microorganisms and Culture Conditions

*Enterococcus faecalis* (ATCC 29212), *Escherichia coli* (ATCC 25922), *Pseudomonas aeruginosa* (ATCC 27853), methicillin-resistant *Staphylococcus aureus* (MRSa; ATTCC 43300), *Staphylococcus epidermidis* (ATCC 12228), *Streptococcus mutans* (ATCC 25175), *Candida albicans* (ATCC 10231), and *Candida glabrata* (ATCC 2001) were thawed and streaked onto an agar surface. Subsequently, a single colony was transferred to culture broth and incubated until an exponential growth phase was reached. The culture was centrifuged, resuspended in phosphate-buffered saline (PBS), and the optical density (OD_620 nm_) of bacterial samples was adjusted to 10^8^ colony-forming units per milliliter (CFU mL^–1^) using a spectrophotometer (Thermo Scientific, Waltham, MA). For yeasts, cell density was assessed using a Neubauer chamber due to morphological variations within the genus. Table S1 indicates the OD_620 nm_ values and culture media used for each microorganism.

### 4.3 Evaluation of the Antimicrobial Activity

The antimicrobial activity of ANO was assessed using agar well diffusion (AWD) and broth microdilution (BM) methods. For the AWD technique, melted agar culture medium (cooled to 45 °C; Table S1) was inoculated with each microorganism (10^6^ CFU mL^–1^). The inoculated medium was then poured into Petri plates containing a previously prepared base agar layer. After solidification, 5 mm diameter wells were placed on the agar surface. Twenty microliters of ANO (200 mg mL^–1^) were added to the wells in triplicate. PBS and chlorhexidine (0.12 vol %) were used as negative and positive controls, respectively. The plates were kept at room temperature for 2 h before incubation at 37 °C for 24 h. The diameters of the inhibition halos were measured and recorded in millimeters.

The BM assay was performed according to the Clinical & Laboratory Standards Institute guidelines.^35^ Briefly, ten 2-fold serial dilutions (from 25 mg mL^–1^ to 0.045 mg mL^–1^) were prepared in 96-well plates (Kasvi). Each well was then inoculated with the test microorganisms (bacteria: 10^5^ CFU mL^–1^; yeast: 10^4^ CFU mL^–1^), in duplicate. Two additional wells were included as controls: a sterility control (without inoculum) and a growth control (without ANO). The plates were incubated at 37 °C for 24 h. The minimum inhibitory concentration (MIC) was determined as the lowest ANO concentration at which no visible microbial growth was observed. To determine the minimum bactericidal concentration (MBC) and minimum fungicidal concentration (MFC), aliquots from wells showing no visible growth were plated onto the agar and incubated under the same conditions.

### 4.4 Evaluation of the Antibiofilm Activity

The antibiofilm activity of ANO was evaluated in mature biofilms, assessing the effects on biomass, metabolic activity (XTT), and structural morphology. The 48 h biofilms were grown on the external surface of 96-well qPCR plates (Thermo Scientific), in triplicate, as described by Doucet et al., with minor modification.^36^ Briefly, 96-well plates received 200 μL of culture broth inoculated at 10^6^ CFU mL^–1^. In each plate, a sterile qPCR plate was introduced, allowing the culture medium to surround the pins of the qPCR plate. Incubation was carried out at 37 °C for 48 h under orbital shaking. After 24 h, half of the medium was removed, and another 100 μL of fresh culture medium was added. After the incubation period, the qPCR plates were removed from the original culture plate and repeatedly transferred three times into other 96-well plates containing sterile PBS to remove the planktonic cells.

To evaluate the minimum biofilm eradication concentration (MBEC), 8 concentrations of ANO ranging from 200 to 1.55 mg mL^–1^ were prepared.^37^ MBEC was defined as the lowest concentration that was able to disrupt the established biofilm layers. Two hundred microliters were then transferred to 96-well plates, and the qPCR plates, with the mature biofilms, were introduced into the different concentrations of ANO. PBS was used as a negative control and 2 vol % sodium hypochlorite as a positive control. Two contact times (10 and 30 min) were evaluated to investigate the antibiofilm activity over time. After the immersion periods, the plates were removed from the wells and rinsed twice with PBS.

To assess metabolic activity, 96-well plates were filled with 158 μL of PBS supplemented with 100 mM glucose (Sigma-Aldrich, St. Louis, MO), 40 μL of XTT at 1 mg mL^–1^ (Sigma-Aldrich), and 2 μL of menadione at 0.4 mM (Sigma-Aldrich).^38^ The qPCR plates, with the mature biofilms, were placed into the wells and incubated, protected from light, at 37 °C for 2 h. Then, the qPCR plates were removed and the absorbance of the resulting solution was measured at 492 nm using a spectrophotometer (Thermo Scientific).

For biomass evaluation, 96-well plates were filled with 200 μL of ethanol (Sigma-Aldrich). The qPCR plates with the mature biofilms were placed into the wells and incubated for 15 min. After solvent removal, the plates were dried, and the biofilms were transferred to other 96-well plates containing 200 μL of 1 wt % crystal violet (Dinamica, Indaiatuba, SP, Brazil). After 5 min of incubation, the plates were washed three times with PBS. Once dried, the plates were immersed in 33 vol % acetic acid (Sigma-Aldrich), in new 96-well plates. The indirect quantification of the total biomass of the biofilms was determined by measuring the absorbance at 570 nm using a spectrophotometer.^37^

For evaluating the biofilm morphology, some pins from the qPCR plates immersed in ANO during 30 min were cut and fixed with 2.5 vol % glutaraldehyde (Sigma-Aldrich) for 24 h. Then, the fragments were dehydrated in a graded ethanol series (30, 50, 70, 90, and 100 vol %) and in hexamethyldisilazane (Sigma-Aldrich). The specimens were fixed on aluminum stubs, coated with gold, and examined at 10,000× magnification under high vacuum using a scanning electron microscope (SEM) (Carl Zeiss, Jena, Germany).^39^

### 4.5 Obtaining the Niobium-Based Conditioning Layer on Titanium Surface

The surfaces of titanium discs (Ø15 × 2 mm) were standardized to a roughness of approximately 0.5 μm (Surftest SJ-201P rugosimeter; Mitutoyo, Tokyo, Japan), through polishing with waterproof sandpapers (3M, Sumaré, SP, Brazil). In the control group, titanium discs (*n* = 30) did not undergo any surface treatment after roughness standardization. The remaining discs had their experimental surface functionalized with ANO films (*n* = 30). Immediately before treatment, each disc was placed in an ultrasonic bath and subjected to the following cleaning protocol: (i) A detergent solution based on 1 wt % sodium dodecyl sulfate (Sigma-Aldrich); (ii) Distilled water; (iii) 70 vol % ethanol; and (iv) Acetone (Sigma-Aldrich). The discs were ultrasonicated for 5 min in each solution.

ANO at 5 mg mL^–1^ and poly­(acrylic acid) (PAA) at 0.1 wt % (Acros Organics, Geel, Belgium) were used to functionalize the titanium surface. The discs were held with hemostatic forceps and immersed in beakers containing the compounds in the following sequence: (i) 0.1 wt % PAA for 6 min; (ii) distilled water for 90 s; (iii) 0.5 wt % ANO for 6 min; (iv) distilled water for 90 s. After each immersion in distilled water, the discs were properly dried using an air pump to allow the deposition of the next layer. Indirect deposition was evaluated after 8, 10, and 12 sequential cycles. The experimental surface was then characterized by ATR-FTIR and gold-coated for SEM analysis (EVO MA10; Carl Zeiss).

### 4.6 Cytotoxicity of ANO and ANO-Based Conditioning Layer

The fibroblast-derived mouse cell line (ATCC L929) was used to determine the cytotoxicity of ANO in eukaryotic cells,^40^ and according to the international standard ISO 10993-12. Two strategies were tested to evaluate cytotoxicity: (i) treatment with ANO (n = 6) and (ii) direct contact with the ANO-based conditioning layer on titanium discs (*n* = 6).

For the treatment with ANO, cells (10^4^ cells well^–1^) were seeded in 96-well plates filled with high glucose Dulbecco’s modified Eagle’s medium (DMEM) (Sigma-Aldrich) supplemented with 10 vol % fetal bovine serum (FBS) (Thermo Scientific). The plates were incubated for 24 h at 37 °C and 5 vol % CO_2_. Then, the culture medium was replaced with fresh medium containing ANO at concentrations of 1.25, 2.5, and 5 mg mL^–1^. The plates were incubated for 24 h more in the presence of ANO. Next, the culture medium was removed, the wells were washed with PBS, and 250 μL of MTT (3-[4,5-methylthiazol-2-yl]-2,5-diphenyl-tetrazolium) (Sigma-Aldrich) at 0.5 mg mL^–1^ was added to each well. Dimethyl sulfoxide (DMSO) (Sigma-Aldrich) was used as a positive control, and wells containing only DMEM medium were used as negative control.

For direct contact with the experimental surface, the specimens were placed in 24-well plates and received 3 × 10^4^ cells in 2 mL of DMEM + 10 vol % FBS as previously described.^41^ Specimens without surface treatment were used as the controls. After 48 h of incubation at 37 °C and 5 vol % CO_2_, the medium was removed, and the wells were washed with PBS. Each well received 800 μL of MTT solution.

The plates, from both strategies, were incubated for 2 h in the dark at 37 °C and 5 vol % CO_2_. Then, the suspension was removed, and the precipitate was solubilized with DMSO. The colorimetric spectrum of the suspension was measured at 570 nm using a spectrophotometer. Relative viability was calculated with respect to the viability of untreated wells.

### 4.7 Antibiofilm Activity of the ANO-Based Conditioning Layer

The antibiofilm effect of the ANO-based conditioning layer was evaluated in a multispecies biofilm model, consisting of MRSA, *P. aeruginosa*, and *C. albicans*. The assay was carried out under dynamic flow conditions to simulate a physiologically relevant environment. The following variables were evaluated: (i) Initial adhesion (area in μm^2^): after 1, 2, and 4 h of flow; (ii) Microbial load (CFU mL^–1^): after 24 h of flow; (iii) Biovolume (area in μm^3^): after 24 h of flow; (iv) Biofilm morphology (SEM): after 24 h of flow. The assay was carried out in 3 independent moments.

The inoculum (bacteria: 10^6^ CFU mL^–1^; yeast: 10^5^ CFU mL^–1^) was prepared in BHI broth supplemented with 5 wt % of bovine serum albumin (Inlab, Diadema, SP, Brazil), as described previously.^26^ Eight specimens from both control and experimental groups were placed in individual chambers, which were maintained at 37 °C throughout the entire experimental period. The flow chambers and bottles, with the inoculated medium, were connected to silicone hoses through a peristaltic pump system. The flow rate was adjusted to 1 mL min^–1^. For up to 4 h of flow (adhesion period), the system was fed with an inoculated medium. After 4 h, up to 24 h (maturation period), the system was fed with sterile culture medium.

After 1, 2, and 4 h, one specimen from each group was removed from the flow chamber and evaluated for microbial adhesion. The specimens were rinsed twice with PBS to remove planktonic cells, stained with viability dyes (Filmtracer LIVE-DEAD Biofilm; Molecular Probes, Eugene, OR) according to the manufacturer’s instructions and observed under fluorescent microscopy (Carl Zeiss). Thirteen fields were evaluated for each specimen, totaling 39 images per group (3 biological replicates), with a magnification of 630×.^42^ The quantification of the microorganisms covered areas (μm^2^) was performed with the aid of the BAIT (Biofilm Architecture Inference Tool) software.^43^

For microbial load assessment, 3 specimens from each group were removed from the chambers and rinsed twice with PBS to remove planktonic cells. Each specimen was transferred to a tube containing 10 mL of Leethen broth (BD Difco, Franklin Lakes, NJ). The tubes were vortexed for 60 s, sonicated (200 W, 40 kHz) for 20 min, and then vortexed again for 2 min to ensure the detachment of all the aggregated biofilm. Serial dilution aliquots (10^–1^ to 10^–7^) were seeded onto selective culture media [*C. albicans*: Chromagar Candida (BD Difco); *P. aeruginosa*: MacConkey Agar (Kasvi); MRSa: Mannitol Salt Agar (Kasvi)] and incubated at 37 °C for 24 h. Subsequently, the number of colonies was registered and expressed as log_10_ CFU mL^–1^.

For biovolume analysis, the specimens were removed from the flow chamber and stained as described for the adhesion test. The specimens were analyzed by confocal laser scanning microscopy (CLSM) (Leica Microsystems, Wetzlar, Germany). Six random fields were evaluated for each specimen, totaling 18 images per group (3 biological replicates).^44^ The series of images obtained on the *Z*-axis were used for 3D reconstruction and measurement of the maximum thickness. The biovolume (μm^3^) was calculated using the height, width, and depth of each voxel (abbreviation for ″volume pixel″) with the aid of the BAIT software.

To evaluate the morphology of the multispecies biofilm grown on both control and ANO-conditioned surfaces, the titanium discs were removed from the flow chamber, rinsed in PBS, and fixed in 2.5 vol % glutaraldehyde (Sigma-Aldrich). Subsequently, the specimens were dehydrated in a series of alcohols, gold-metalized, and analyzed under a SEM.^39^

### 4.8 Statistical Analysis

The data set from the evaluation of the antimicrobial activity and antibiofilm activity of ANO (MIC/MBC/MBEC assays) was analyzed using a descriptive approach based on measures of central tendency and dispersion (mean and standard deviation). MIC/MBC/MBEC values represent predefined biological thresholds rather than comparative measurements; therefore, statistical analyses were not applied, consistent with standard susceptibility testing guidelines.

The variables related to the evaluation of the cytotoxicity and antifouling activity of the ANO-based conditioning layer were categorized as continuous quantitative variables. Therefore, the data set was assessed for normality using the Shapiro-Wilk test as well as for homogeneity of variances using Levene’s test. Given these assumptions, microbial load on the control and experimental surfaces was compared using the Student’s *t* test. Cytotoxicity and biovolume were analyzed using the Mann–Whitney test or Kruskal–Wallis test (Dunn post hoc test with Bonferroni adjustment). The areas covered by cells after 1, 2, and 4 h were analyzed by using a Generalized Linear Model with multiple comparisons and Bonferroni adjustment. All statistical tests were performed using IBM SPSS 25.0 software (IBM Corp., SPSS Statistics for Windows, Version 21.0, Armonk, NY), with a significance level of 0.05.

## Supplementary Material



## Data Availability

The data set generated or analyzed, which supports the main findings of this study, is available in the USP repository (https://repositorio.uspdigital.usp.br/handle/item/840).

## Notes

Description of microorganisms and culture conditions, and details on the experimental setup for microbiological assays (PDF)

## References

[1] CBMM, Companhia Brasileira de Metalurgia e Mineração . Niobium Materials Technology - Niobium-containing superalloys. https://niobium.tech/en/landing-pages/about-niobium/about-niobium (accessed 05 May 2021).

[2] Chézeau L., Tchinda A., Pierson G., Bravetti P., Ferrari L., Joubert O., Zaiou M., Rihn B. H. (2022). *In vitro* molecular
study of titanium-niobium alloy biocompatibility. Biomedicines.

[3] Rajan S. T., Das M., Arockiarajan A. (2022). Biocompatibility
and corrosion evaluation of niobium
oxide coated AZ31B alloy for biodegradable implants. Colloids Surf. B.

[4] Dsouki N. A., de Lima M. P., Corazzini R., Gáscon T. M., Azzalis L. A., Junqueira V. B., Feder D., Fonseca F. L. (2014). Cytotoxic,
hematologic and histologic effects of niobium pentoxide in Swiss mice. J. Mater. Sci. Mater. Med..

[5] Oliveira L., Pereira M., Pacheli
Heitman A., Filho J., Oliveira C., Ziolek M. (2023). Niobium: The
focus on catalytic application in the
conversion of biomass and biomass derivatives. Molecules.

[6] Carneiro K. K., Araujo T. P., Carvalho E. M., Meier M. M., Tanaka A., Carvalho C. N., Bauer J. (2018). Bioactivity
and properties of an
adhesive system functionalized with an experimental niobium-based
glass. J. Mech. Behav. Biomed. Mater..

[7] Balbinot G. d. S., Leitune V. C. B., Ponzoni D., Collares F. M. (2019). Bone healing
with niobium-containing bioactiveglass composition in rat femur model:
A micro-CT study. Dent. Mater..

[8] Chen J., Liu Y., Cheng G., Guo J., Du S., Qiu J., Wang C., Li C., Yang X., Chen T., Chen Z. (2022). Tailored hydrogel delivering
niobium carbide boosts ROS-scavenging
and antimicrobial activities for diabetic wound healing. Small.

[9] Ren J., Da J., Wu W., Zheng C., Hu N. (2022). Niobium carbide-mediated
photothermal therapy for infected wound treatment. Front. Bioeng. Biotechnol..

[10] Altmann A. S. P., Collares F. M., Leitune V. C., Arthur R. A., Takimi A. S., Samuel S. M. (2017). *In vitro* antibacterial and remineralizing
effect of adhesive containing triazine and niobium pentoxide phosphate
inverted glass. Clin. Oral Investig..

[11] Degrazia F. W., Altmann A. S. P., Ferreira C. J., Arthur R. A., Leitune V. C. B., Samuel S. M. W., Collares F. M. (2019). Evaluation
of an antibacterial orthodontic
adhesive incorporated with niobium-based bioglass: An *in situ* study. Braz. Oral Res..

[12] Vaidya M., Mcbain A. J., Banks C. E., Whitehead K. A. (2019). Single
and combined antimicrobial efficacies for nine metal ion solutions
against *Klebsiella pneumoniae*, Acinetobacter baumannii
and Enterococcus faecium. Int. Biodeterior.
Biodegrad..

[13] Baena M. I., Márquez M. C., Matres V., Botella J., Ventosa A. (2006). Bactericidal
activity of copper and niobium-alloyed austenitic stainless steel. Curr. Microbiol..

[14] Senocak T. C., Ezirmik K. V., Cengiz S. (2022). The antibacterial properties
and
corrosion behavior of silver-doped niobium oxynitride coatings. Mater. Today Commun..

[15] Senocak T. C., Ezirmik K. V., Aysin F., Ozek N. S., Cengiz S. (2021). Niobium-oxynitride
coatings for biomedical applications: Its antibacterial effects and
in-vitro cytotoxicity. Mater. Sci. Eng. C: Mater.
Biol. Appl..

[16] Akshaya S., Rowlo P. K., Dukle A., Nathanael A. J. (2022). Antibacterial
coatings for titanium implants: Recent trends and future perspectives. Antibiotics.

[17] Xie Y., Pan Y., Cai P. (2022). Cellulose-based
antimicrobial films incroporated with
ZnO nanopillars on surface as biodegradable and antimicrobial packaging. Food Chem..

[18] Li S., Meng L., Zhu Y., Zhang W., Sun Y., Bai G., Li X. (2023). Copper ion-loaded
surface charge-convertible coatings
on implant: Antibacterial and tunable cell adhesion properties. Chem. Eng. J..

[19] Pereira, A. T. ; Oliveira, K. A. ; Souza, R. Processo de produção de oxalato amoniacal de nióbio, oxalato amoniacal de nióbio e uso do mesmo. Companhia Brasileira de Metalurgia e Mineração. PI0403891-6A2, 2004.

[20] Su T. T., Zhai Y., Jiang H., Gong H. (2009). Studies on the thermal
decomposition kinetics and mechanism of ammonium niobium oxalate. J. Therm. Anal. Calorim..

[21] Vincent J. L., Sakr Y., Singer M., Martin-Loeches I., Machado F. R., Marshall J. C., Finfer S., Pelosi P., Brazzi L., Aditianingsih D., Timsit J. F., Du B., Wittebole X., Máca J., Kannan S., Gorordo-Delsol L. A., De Waele J. J., Mehta Y., Bonten M. J. M., Khanna A. K., Kollef M., Human M., Angus D. C. (2020). EPIC III Investigators.
Prevalence and outcomes of infection among patients in intensive care
units in 2017. JAMA.

[22] Raoofi S., Pashazadeh Kan F., Rafiei S., Hosseinipalangi Z., Noorani Mejareh Z., Khani S., Abdollahi B., Seyghalani Talab F., Sanaei M., Zarabi F., Dolati Y., Ahmadi N., Raoofi N., Sarhadi Y., Masoumi M., Sadat Hosseini B., Vali N., Gholamali N., Asadi S., Ahmadi S., Ahmadi B., Beiramy
Chomalu Z., Asadollahi E., Rajabi M., Gharagozloo D., Nejatifar Z., Soheylirad R., Jalali S., Aghajani F., Navidriahy M., Deylami S., Nasiri M., Zareei M., Golmohammadi Z., Shabani H., Torabi F., Shabaninejad H., Nemati A., Amerzadeh M., Aryankhesal A., Ghashghaee A. (2023). Global prevalence of nosocomial infection: A systematic
review and meta-analysis. PLoS One.

[23] European Centre for Disease Prevention and Control . Healthcare-Associated Infections Acquired in Intensive Care Units; ECDC: Stockholm, 2024.

[24] Dizaj S. M., Lotfipour F., Barzegar-Jalali M., Zarrintan M. H., Adibkia K. (2014). Antimicrobial activity of the metals
and metal oxide
nanoparticles. Mater. Sci. Eng. C: Mater. Biol.
Appl..

[25] Frei A., Verderosa A. D., Elliott A. G., Zuegg J., Blaskovich M. A. T. (2023). Metals
to combat antimicrobial resistance. Nat. Rev.
Chem..

[26] Faot F., Cavalcanti Y. W., Mendonça-Bertolini M., Pinto L. R., da Silva W. J., Cury A. A. (2014). Efficacy of citric acid denture cleanser
on the *Candida albicans* biofilm formed on poly­(methyl
methacrylate): effects on residual biofilm and recolonization process. BMC Oral Health.

[27] Khalid M. U., Rudokaite A., da Silva A. M. H., Kirsnyte-Snioke M., Stirke A., Melo W. C. M. A. (2025). A
comprehensive review of niobium
nanoparticles: synthesis, characterization, applications in health
sciences, and future challenges. Nanomaterials.

[28] Kolya H., Kang C. W. (2024). Toxicity of metal
oxides, dyes, and dissolved organic
matter in water: Implications for the environment and human health. Toxics.

[29] Zheng S., Bawazir M., Dhall A., Kim H. E., He L., Heo J., Hwang G. (2021). Implication
of surface properties, bacterial motility,
and hydrodynamic conditions on bacterial surface sensing and their
initial adhesion. Front. Bioeng. Biotechnol..

[30] Jiang P., Zhang Y., Hu R., Shi B., Zhang L., Huang Q., Yang Y., Tang P., Lin C. (2023). Advanced surface
engineering of titanium materials for biomedical applications: From
static modification to dynamic responsive regulation. Bioact. Mater..

[31] Liufu S., Xiao H., Li Y. (2005). Adsorption
of poly­(acrylic acid)
onto the surface of titanium dioxide and the colloidal stability of
aqueous suspension. J. Colloid Interface Sci..

[32] Safavi M. S., Khalil-Allafi J., Restivo E., Ghalandarzadeh A., Hosseini M., Dacarro G., Malavasi L., Milella A., Listorti A., Visai L. (2023). Enhanced *in vitro* immersion behavior and antibacterial activity of
NiTi orthopedic
biomaterial by HAp-Nb2O5 composite deposits. Sci. Rep..

[33] Souza L. V. S., Pavanello L., Picolo M. Z. D., Kury M., Matos I. C. R. T., Cogo-Müller K., Esteban Florez F. L., Cavalli V. (2023). Mechanical and antibacterial properties of an experimental
flowable composite containing Nb2O5 and NF_TiO2 nanoparticles. J. Mech. Behav. Biomed. Mater..

[34] Detomaso L., Gristina R., Senesi G. S., d’Agostino R., Favia P. (2005). Stable plasma-deposited acrylic acid
surfaces for cell culture applications. Biomaterials.

[35] Methods for Dilution Antimicrobial Susceptibility Tests for Bacteria That Grow Aerobically, 12th ed.; Clinical and Laboratory Standards Institute: Wayne, 2024.

[36] Doucet A. N., Slipski C. J., Golding G. R., Mulvey M. R., Bay D. C. (2022). Generation
of greater bacterial biofilm biomass using PCR-Plate deep well microplate
devices. J. Vis. Exp..

[37] Wijesinghe G. K., Feiria S. B., Maia F. C., Oliveira T. R., Joia F., Barbosa J. P., Boni G. C., HÖfling J. F. (2021). *In vitro* antibacterial and antibiofilm
activity of *Cinnamomum verum* leaf oil against *Pseudomonas aeruginosa*, *Staphylococcus aureus* and *Klebsiella pneumoniae*. An. Acad. Bras. Cienc..

[38] Silva W. J. d., Seneviratne J., Parahitiyawa N., Rosa E. A., Samaranayake L. P., Del Bel Cury A. A. (2008). Improvement
of XTT assay performance for studies involving *Candida albicans* biofilms. Braz. Dent.
J..

[39] Oliveira V. C., Macedo A. P., Melo L. D. R., Santos S. B., Hermann P. R. S., Silva-Lovato C. H., Paranhos H. F. O., Andrade D., Watanabe E. (2021). Bacteriophage cocktail-mediated
inhibition of *Pseudomonas aeruginosa* biofilm on endotracheal
tube surface. Antibiotics.

[40] Kim S., Lee D. W., Jin J. S., Kim J. (2020). Antimicrobial activity
of LysSS, a novel phage endolysin, against *Acinetobacter baumannii* and *Pseudomonas aeruginosa*. J. Glob. Antimicrob. Resist..

[41] Podgórski R., Wojasiński M., Ciach T. (2022). Nanofibrous materials
affect the
reaction of cytotoxicity assays. Sci. Rep..

[42] Marques D. M., Oliveira V. C., Souza M. T., Zanotto E. D., Issa J. P. M., Watanabe E. (2020). Biomaterials for orthopedics:
Anti-biofilm activity
of a new bioactive glass coating on titanium implants. Biofouling.

[43] Luo T. L., Hayashi M., Zsiska M., Circello B., Eisenberg M., Gonzalez-Cabezas C., Foxman B., Marrs C. F., Rickard A. H. (2019). Introducing
BAIT (Biofilm Architecture Inference Tool): a software program to
evaluate the architecture of oral multi-species biofilms. Microbiology.

[44] Raile P. N., Oliveira V. C., Macedo A. P., Curylofo P. A., Marcato P. D., Watanabe E., Paranhos H. F. O., Pagnano V. O. (2023). Action of chitosan-based
solutions against a model four-species biofilm formed on cobalt-chromium
and acrylic resin surfaces. Gerodontology.

