# Supplementary material for: Niobium-Based Conditioning Layer to Reduce Bacterial Adhesion and Biofilm Formation on Titanium Surface
Source: ACS Omega. 2026 Mar 26;11(13):20782–94. doi: 10.1021/acsomega.5c12932 (PMC13063182; doi:10.1021/acsomega.5c12932)
Supplement: Supplementary file 1 [file ao5c12932_si_001.pdf]

Supporting Information

Niobium-based conditioning layer to reduce bacterial adhesion and biofilm formation on titanium surface

Viviane C. Oliveira[a,b], Nilza L. Magalhães[c,d], Carla R.O. Maciel[a], André F.A.S. Silva[e], Lucas L. Bim[f], Carolina Chaves[g], Cássio do Nascimento[a], Francisco W. Paula-Silva[c], Cláudia H. Silva-Lovato[a], Ana P. Ramos[e], Adriano M. Ferreira[h], Evandro Watanabe[i,*]

Table S1: Microorganisms and culture conditions.

Microorganisms	Optical density (10^8 CFU/mL)	Culture media for AWD, BM and microbial load	
		**Broth**	**Agar**
E. faecalis	0.130 – 0.150	Mueller Hinton (MH) (Kasvi, São José dos Pinhais, PR, Brazil)	Agar MH
E. coli	0.100 – 0.110	MH	Agar MH
P. aeruginosa	0.100 – 0.110	MH	Agar MH
MRSa	0.090 – 0.100	MH	Agar MH
S. epidermidis	0.090 – 0.100	MH	Agar MH
S. mutans	0.150 – 0.180	Brain Heart Infusion (BHI) (Kasvi)	Agar BHI
C. albicans	-	Sabouraud Dextrose (SD) (Kasvi)	Agar SD
C. glabrata	-	SD	Agar SD

MRSa: methicillin resistant *Staphylococcus aureus*; MH: Mueller Hinton; BHI: Brain Heart Infusion; SD: Sabouraud Dextrose.
